# Study on Thermal Effect of Aluminum-Air Battery

**DOI:** 10.3390/nano13040646

**Published:** 2023-02-06

**Authors:** Yajun Cai, Yunwei Tong, Yingjie Liu, Xinyu Li, Beiyang Chen, Feng Liu, Baowei Zhou, Yichun Liu, Zhenbo Qin, Zhong Wu, Wenbin Hu

**Affiliations:** 1 School of Materials Science and Engineering, Tianjin Key Laboratory of Composite and Functional Materials, Key Laboratory of Advanced Ceramics and Machining Technology (Ministry of Education), Tianjin University, Tianjin 300072, China; 2 Huadian Water Technology Co., Ltd., Beijing 100160, China; 3 China Huadian Co., Ltd., Beijing 100031, China; 4 School of Materials Science and Engineering, Kunming University of Science and Technology, Kunming 650093, China; 5 Joint School of National University of Singapore and Tianjin University, International Campus of Tianjin University, Fuzhou 350207, China

**Keywords:** aluminum-air battery, thermal effect, hydrogen-evolution reaction, hybrid additives

## Abstract

The heat released from an aluminum-air battery has a great effect on its performance and operating life during the discharge process. A theoretical model was proposed to evaluate the resulting thermal effect, and the generated heat was divided into the following sources: anodic aluminum oxidation reaction, cathodic oxygen reduction reaction, heat production against the battery internal resistance, and hydrogen-evolution reaction. Quantitative analysis was conducted on each part, showing that all heat production sources increased with discharge current density. It should be noted that the heat caused by hydrogen evolution accounted for the most, up to 90%. Furthermore, the regulation strategy for inhibiting hydrogen evolution was developed by addition of hybrid additives to the electrolyte, and the hydrogen-evolution rate was greatly reduced by more than 50% as was the generated heat. This research has important guidance for the thermal effect analysis of aluminum–air batteries, together with control of the thermal management process by inhibiting hydrogen evolution, thus promoting their practical application.

## 1. Introduction

With rapid construction of clean energy networks and development of new energy industries such as photovoltaic, wind power, and electrical vehicles, modern society has put forward higher requirements for energy density and security of battery energy systems [1,2]. Among the newly proposed electrochemical devices, metal-air batteries are a promising alternative to lithium-based batteries. The most important feature of a metal–air battery is a metal anode coupling characterized by high-energy density, together with an open-structure-catalyzed cathode that continuously draws oxygen from the atmosphere. This feature is responsible for the intrinsically high theoretical energy density generally associated with this type of device [3]. Aqueous aluminum-air batteries show great potential due to their high theoretical electrochemical capacity (2980 mAh·g^−1^ [4,5]) and specific energy (8100 Wh·kg^−1^ [6,7]), the richest Earth reserves with low cost (8.21 wt. %) [8], and intrinsic safety (noncombustible). Therefore, aluminum-air batteries have been recognized as an attractive candidate for next-generation batteries [5]. The choice of electrolytes used in aluminum-air batteries is fairly flexible. The electrolyte can be common salt (NaCl), sea water, or alkaline electrolytes such as sodium hydroxide (NaOH) [9,10]. Alkaline aqueous solution was usually applied as its electrolyte to achieve high conductivity and to eliminate surface passivation of the metal anode [11]. The corresponding discharge reactions consist of an aluminum oxidation reaction at the anode, an oxygen reduction reaction (ORR) at the cathode, and a side reaction of hydrogen evolution. The specific reaction equations (Equations (1)–(4)) follow:

(1) $$\mathrm{Anode}\;\mathrm{reaction}:\;Al-3e^{-}\to Al^{3+}$$

(2) $$\mathrm{Cathode}\;\mathrm{reaction}:\;O_{2}+2H_{2}O+4e^{-}\to 4OH^{-}$$

(3) $$\mathrm{Total}\;\mathrm{reaction}:4Al+3O_{2}+6H_{2}O=4Al{\left(OH\right)}_{3}$$

(4) $$\mathrm{Side}\;\mathrm{reaction}:Al+3H_{2}O+OH^{-}=3/2H_{2}+Al{\left(OH\right)}_{4}^{-}$$

The thermal effect of batteries could significantly affect their performance and operating life, resulting in serious safety issues, such as electrolyte decomposition [12] and thermal runaway of the battery [13,14]. Therefore, it is necessary to evaluate the thermal characteristics, and a series of related research has been conducted on lithium batteries. Generally, the heat generated by a lithium-ion battery could be divided into four parts consisting of reaction heat induced by Li^+^ transfer (*Q_R_*), joule heat against ohmic internal resistance (*Q_J_*), polarization heat from the electrode polarization effect (*Q_P_*), and side-reaction heat caused by the self-discharge reaction of the battery (*Q_S_*) [15,16,17]. Further, the proportion of the abovementioned parts was investigated at different discharge rates [18], which indicated that the heat production of the battery was mainly joule heat at high discharge rates, while reaction heat accounted for the majority at low rates. Furthermore, a heat generation model of lithium-ion batteries was established by coupling electrochemical principles to verify the accuracy of the model through the temperature rise under different charge and discharge rates [19]. The thermal behavior of bagged lithium-ion batteries at different charging and discharging rates (0.5-1-2 C) was studied at operating temperatures of 30, 40, and 54 °C [20]. It was shown that the rates of charge and discharge inversely affect battery performance. In addition, the heat generation equation of the battery system was also proposed according to the law of energy conservation, which has been used to predict battery discharge performance at different operating temperatures [21]. Various studies have been carried out to explore the heating mechanism of lithium-ion batteries and established the relationship between performance and operating temperature. Obviously, evaluating the thermal effect of aluminum-air batteries is crucial for their practical application. However, there has been no relevant study on aluminum-air batteries until now.

**Figure 1:**
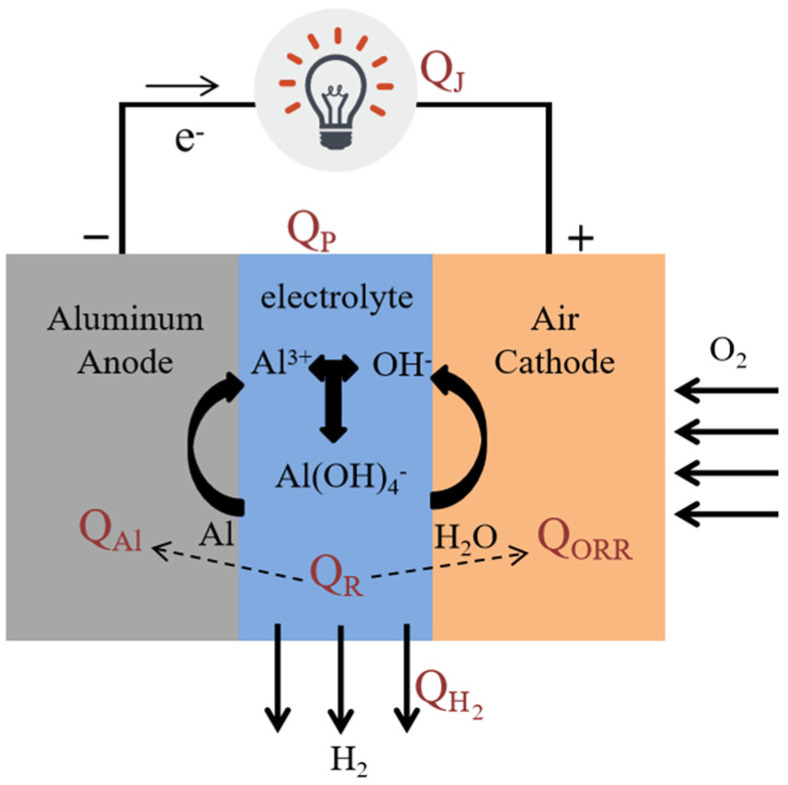
Schematic diagram showing the evaluation model for the thermal effect of an aluminum-air battery.

Owing to the similar discharge principle of a metal battery, an evaluation model for the thermal effect of an aluminum–air battery was proposed, referring to lithium-ion battery [15]. The corresponding schematic diagram is exhibited in Figure 1, including *Q_R_* (referring to the discharge reaction consisting of aluminum oxidation at the anode, *Q_Al_* [18], and the oxygen reduction reaction at the cathode, *Q_ORR_* [22]), *Q_J_* (joule heat against ohmic internal resistance of the aluminum-air battery) [18,22], *Q_P_* (polarization heat from the electrode polarization effect) [23], and *Q*_*H*_2__ [22] (referring to the side reaction of hydrogen evolution). The specific values for each part and their changing trends with discharge rates were evaluated quantitatively in this work, conducive to establishing a certain data model and theoretical basis for the thermal effect of aluminum-air batteries.

Moreover, the strategy for solving thermal runaway of the battery was also provided to optimize the thermal management process and subsequently promote large-scale commercial application of aluminum-air batteries. Currently, two main strategies have been reported to reduce thermal runaway in batteries. The first strategy is aluminum anode alloying. Alloying aluminum with specific elements is one of the effective ways to reduce the oxidation overpotential of aluminum in an alkaline medium, inhibit the rate of parasitic corrosion, and reduce thermal runaway. For example, adding Sb [24], Mg [25], Mn [26], or Li [27] to pure aluminum alloy with high hydrogen overpotential can inhibit hydrogen evolution, thus reducing thermal runaway of the battery and improving the safety of the battery. However, most alloying elements occur as a solid solution in aluminum, which performs an activation or corrosion inhibition role. The electrochemical performance of the aluminum anode will be affected if the alloying elements precipitate and form a second phase at the grain boundary. The other strategy is to control the side reaction of the aluminum-air battery by electrolyte modification, so as to control the thermal runaway problem. For example, using polyvinyl alcohol (PVA) as the anode liquid and neutral salt xanthan gum as the cathode liquid, dual electrolyte (DE) aluminum-air batteries were prepared without a separator. In this system, the hydrogen-evolution reaction can be reduced by decreasing the water content of the anode, thus controlling the thermal runaway of the battery [28]. We modulated the thermal effect by adding additives directly to the aluminum-air battery electrolyte, which is a more common strategy for scaled applications.

## 2. Experimental

### 2.1. Construction of Thermal Effect Test System

**Figure 2:**
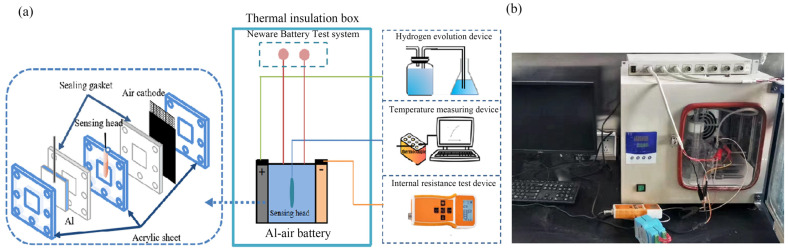
Schematic diagram (**a**) and optical photo (**b**) of the aluminum-air battery structure and thermal effect test system.

A self-designed thermal effect test system was assembled as shown in Figure 2. The experimental environment temperature was controlled at 25 °C ± 1 °C. The battery was subjected to constant-current discharge tests using a Neware battery test system (CT-3008-5V3A-164, Neware Technology Limited, Shenzhen, China). The distance between the anode (pure aluminum, 99.9 wt. %) and air electrode consisting of commercial MnO_2_/C catalyst is 1 cm with 4 M NaOH solution as the electrolyte [28,29]. Prior to testing, the aluminum electrode, with an active area of 50 mm × 50 mm, was abraded with silicon carbide paper and then rinsed with deionized water.

The discharge current densities were 2.5, 5, 10, 15, and 20 mA cm^−2^, with discharge time of 1 h. The total heat released (*Q_tol_*) during the discharge process was assessed directly by a temperature measuring device (test accuracy of ±0.1°C), while *Q_J_* and *Q_P_* were determined by an internal resistance test device (M2111, Smacq, Beijing, China) and *Q*_*H*_2__ by a hydrogen-evolution test device. In addition, the sample was weighed to obtain weight loss before and after discharge.

### 2.2. Hydrogen-Evolution Test

The hydrogen-evolution rate was calculated by collecting H_2_ gas volume at different discharge densities by the water drainage method. All the measurements were repeated at least three times in order to achieve convincing results. The equation used for calculating the hydrogen-evolution rate (Equation (5)) was obtained by referring to previous studies [30]:

(5) $$R=\frac{V_{H_{2}}}{A\times t}$$

where *R* is the hydrogen-evolution rate (mL·cm^−2^·h^−1^), *V*_*H*_2__ is the volume of hydrogen (mL), *A* is the working area of the aluminum anode (cm^2^); and *t* is discharge time (1 h).

### 2.3. Internal Resistance Test of Battery

The internal resistance of the battery (*R_int_*) consisted of ohmic resistance (*R_ohm_*) and polarization resistance (*R_p_*), based on previous research by Pastor-Fernandez et al. [30]. To evaluate the values of *Q_J_* and *Q_P_*, the *R_int_* of the aluminum-air battery in this work was measured by a battery internal resistance meter (RC3563, Hopetcsh, Nanjing, China) with a sampling accuracy of 0.01 Ω.

### 2.4. Specific Heat Capacity Test of the Electrolyte

Differential scanning calorimetry (Q2000, TA, New Castle, DE, USA) was used to determine the specific heat capacity of the electrolyte at different temperatures. The test was conducted within the temperature range 16−90 °C with a heating rate of 5 °C/min and a sampling accuracy of 0.1 °C.

### 2.5. Electrolyte Additives

Hybrid additives were introduced into the electrolyte to regulate the hydrogen-evolution reaction and the thermal effect of the battery. Dodecyl trimethyl ammonium bromide (DTAB, 0.16 M) and ZnO (0.2 M) were added to the basic electrolyte of 4 M NaOH.

## 3. Results and Discussion

### 3.1. Composition of Heat Release during the Discharge Process

#### 3.1.1. *Q_tol_*

The total heat release (*Q_tol_*) of the aluminum-air battery during the discharge process could be directly measured by monitoring temperature change (∆*T*) during discharge, and then calculated by Equation (6):

(6) $$Q_{tol}=c\;m\;\Delta T$$

where *c* is the specific heat capacity of battery electrolyte (J/g·°C), *m* is the mass of electrolyte (g), and ∆*T* is the temperature difference of electrolyte before and after discharging for 1 h (°C). The formula shows that the total heat release depends on the specific heat capacity of the battery electrolyte and the temperature difference between the charge−discharge processes for the aluminum-air battery. It should be noted that the specific heat capacity of the electrolyte varied with temperature, and thus the integral form of Equation (6) could be expressed as Equation (7). T_1_ and T_2_ corresponded to the electrolyte temperature before and after discharge, respectively.

(7) $$Q_{tol}=\int_{T1}^{T2}c\;m\;\mathrm{dT}$$

According to the thermal effect theory of a lithium-ion battery [15,31], *Q_tol_* of the aluminum-air battery can also be expressed as Equation (8):

(8) $$Q_{tol}=Q_{R}+Q_{J}+Q_{P}+Q_{H_{2}}$$

#### 3.1.2. *Q_R_*

*Q_R_* refers to the heat released from the discharge reaction, which consists of heat generated by the aluminum oxidation reaction (*Q_Al_*) and the oxygen reduction reaction (*Q_ORR_*). The calculation of *Q_Al_* is mainly based on the enthalpy change in the aluminum electrode reaction, and the corresponding reaction formula and enthalpy change are expressed as Equations (9) and (10), respectively:

(9) $$Al-3e^{-}\to Al^{3+}$$

(10) $$\Delta_{r}H_{m}^{\theta }\left(298\;K,Al\right)=\Delta_{f}H_{m}^{\theta }\left[Al^{3+}\right]-\Delta_{f}H_{m}^{\theta }\left[Al\right]$$

According to Faraday’s Law, the amount of electricity consumed can be used to represent the amount of reactant n_r_ [32]:

(11) $$n_{r}=It/F$$

where *I* is the current density of the battery discharge (A) and *F* is the Faraday constant (96,485 C·mol^−1^). However, the change in molar enthalpy is no longer a fixed value during reaction process. According to Kirchhoff’s formula [33], the change in enthalpy for the reaction at different temperatures can be obtained by using Equation (12):

(12) $$\Delta_{r}H_{m}^{\theta }\left(T,Al\right)=\Delta_{r}H_{m}^{\theta }\left(298\;K,Al\right)+\overset{T}{\underset{298}{\int }}\sum^{\;}V_{B}\Delta C_{p,m}^{Al}\left(B\right)dT$$

where *V_B_* is the stoichiometric number and $\Delta C_{p,m}^{Al}\left(B\right)$ is the specific heat capacity at constant pressure of the substance. The specific value can be found in Table 1. Equation (12) can then be written as follows:

(13) $$\Delta_{r}H_{m}^{\theta }\left(T\right)=\Delta_{r}H_{m}^{\theta }\left(298\;K,Al\right)+\Delta C_{p,m}^{Al}\;\left(T-298\right)$$

where $\Delta C_{p,m}^{Al}\left(B\right)$ can be expressed as Equation (14):

(14) $$\Delta C_{p,m}^{Al}=C_{p,m}\left[Al^{3+}\right]-C_{p,m}\left[Al\right]$$

*Q_Al_* can be expressed as Equation (15) [14]:

(15) $$Q_{Al}=n_{r}\;\Delta_{r}H_{m}^{\theta }\left(T\right)$$

(16) $$Q_{Al}=It/F\cdot \left\{\Delta_{f}H_{m}^{\theta }\left[Al^{3+}\right]-\Delta_{f}H_{m}^{\theta }\left[Al\right]+\left(C_{p,m}\left[Al^{3+}\right]-C_{p,m}\left[Al\right]\right)\;\left(T-298\right)\right\}$$

Similarly, the Q_ORR_ calculation is also based on the enthalpy change in ORR at the cathode, and the cathodic reaction and standard enthalpy change are shown as Equations (17) and (18), respectively:

(17) $$O_{2}+2H_{2}O+4e^{-}\to 4OH^{-}\;$$

(18) $$\Delta_{r}H_{m}^{\theta }\left(298\;K,ORR\right)=4\;\Delta_{f}H_{m}^{\theta }[\left[OH^{-}\right]-\Delta_{f}H_{m}^{\theta }\left[O_{2}\right]-2\;\Delta_{f}H_{m}^{\theta }\left[H_{2}O\right]]$$

The enthalpy change at different temperatures can be expressed as Equation (19):

(19) $$\Delta_{r}H_{m}^{\theta }\left(T,ORR\right)=\Delta_{r}H_{m}^{\theta }\left(298\;K,ORR\right)+\Delta C_{p,m}^{ORR}\;\left(T-298\right)$$

where $\Delta C_{p,m}^{ORR}$ can be expressed as Equation (20):

(20) $$\Delta C_{p,m}^{ORR}=4\;\Delta_{f}H_{m}^{\theta }[\left[OH^{-}\right]-\Delta_{f}H_{m}^{\theta }\left[O_{2}\right]-2\;\Delta_{f}H_{m}^{\theta }\left[H_{2}O\right]]$$

Q_ORR_ can be expressed as:

(21) $$Q_{ORR}=n_{r}\;\Delta_{r}H_{m}^{\theta }\left(T\right)$$

(22) $$Q_{ORR}=It/F\cdot \{4\;\Delta_{f}H_{m}^{\theta }[\left[OH^{-}\right]-\Delta_{f}H_{m}^{\theta }\left[O_{2}\right]-2\;\Delta_{f}H_{m}^{\theta }\left[H_{2}O\right]]+(4\;\Delta_{f}H_{m}^{\theta }\left[\left[OH^{-}\right]-\Delta_{f}H_{m}^{\theta }\left[O_{2}\right]-2\;\Delta_{f}H_{m}^{\theta }\left[H_{2}O\right]]\right)\left(T-298\right)\}$$

#### 3.1.3. *Q_J_* and *Q_P_*

*Q_J_* is the heat generated by ohm resistance when current flows through the aluminum-air battery, which is subject to Joule’s law and can be expressed as Equation (23):

(23) $$Q_{J}=I^{2}R_{ohm}$$

*Q_P_* is the polarization heat induced by the electrode polarization effect, and the specific value can be achieved by Equation (24):

(24) $$Q_{P}=I^{2}R_{P}$$

According to Pastor-Fernandez et al. [30], it can be shown that the internal resistance of the battery (*R_int_*) consists of ohmic resistance (*R_ohm_*) and polarization resistance (*R_p_*). Additionally, *R_p_* always changes dynamically and is difficult to monitor; thus, *R_int_* is measured directly to investigate the sum of *Q_J_* and *Q_P_* instead of quantitative analysis of them separately. The corresponding *Q_int_* is calculated by Equation (25) [34,35]:

(25) $$Q_{int}=I^{2}R_{int}$$

#### 3.1.4. *Q*_*H*_2__

The side reaction of the aluminum–air battery is mainly a process of hydrogen evolution and *Q*_*H*_2__ is also calculated based on the enthalpy change in the hydrogen-evolution reaction. The detailed reaction formula and standard enthalpy change are expressed as Equations (26)–(28):

(26) $$Al+3H_{2}O+OH^{-}=3/2H_{2}+Al{\left(OH\right)}_{4}^{-}$$

(27) $$\Delta_{r}H_{m}^{\theta }\left(298\;K,H_{2}\right)=\frac{3}{2}\Delta_{f}H_{m}^{\theta }\left[H_{2}\right]+\Delta_{f}H_{m}^{\theta }\left[Al{\left(OH\right)}_{4}^{-}\right]-\Delta_{f}H_{m}^{\theta }\left[Al\right]-3\;\Delta_{f}H_{m}^{\theta }\left[H_{2}O\right]-\Delta_{f}H_{m}^{\theta }\left[OH^{-}\right]$$

(28) $$\Delta_{r}H_{m}^{\theta }\left(T,H_{2}\right)=\Delta_{r}H_{m}^{\theta }\left(298\;K\right)+\Delta C_{p,m}^{H_{2}}\;\left(T-298\right)$$

where $\Delta C_{p,m}^{H_{2}}$ can be expressed as Equation (29):

(29) $$\Delta C_{p,m}^{H_{2}}=3/2C_{p,m}\left[H_{2}\right]+C_{p,m}\left[Al{\left(OH\right)}_{4}^{-}\right]-3C_{p,m}\left[H_{2}O\right]-C_{p,m}\left[Al\right]-C_{p,m}\left[OH^{-}\right]$$

The amount of H_2_ (mol) produced in unit time t (h) is obtained by Equation (30):

(30) $$n_{H_{2}}=V_{H_{2}}/22.4$$

The heat generated by the hydrogen-evolution reaction, *Q*_*H*_2__, is expressed by Equation (31):

(31) $$Q_{H_{2}}=\Delta_{r}H_{m}^{\theta }\left(T,H_{2}\right)\cdot V_{H_{2}}/22.4$$

#### 3.1.5. *Q_A_*

At the aluminum anode, the discharge reaction occurs simultaneously with the side reaction of hydrogen evolution, and they both consumed electrons released by the dissolution of aluminum electrode. The heat generated by the aluminum anode is introduced as *Q_A_*, which obviously consists of *Q_Al_* and *Q*_*H*_2__. It can be determined by the mass-loss method [35,36] and calculated by Equation (15) with *n_r_* referred with *n_A_*, based on Equation (32):

(32) $$n_{A}=m_{Al\;}/M_{Al}$$

where *m_Al_* is recorded as the weight loss before and after the discharge reaction (g), and *M_Al_* is the molar mass of the aluminum electrode (g/mol). Therefore, the aluminum anode heat production *Q_A_* can also be expressed as Equation (33):

(33) $$Q_{A}=Q_{Al}+Q_{H_{2}}$$

### 3.2. Quantitative Analysis for Heat Generated by Each Part

**Figure 3:**
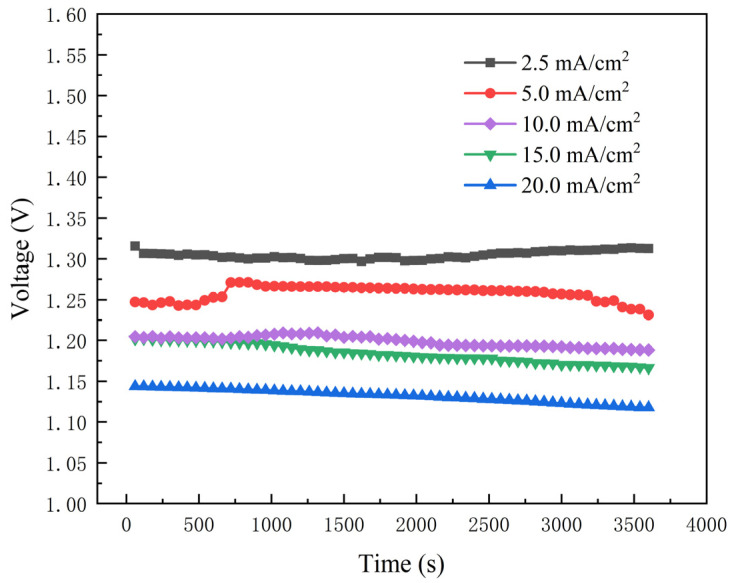
The discharge curves for the aluminum-air battery at different discharge current densities in 4 M NaOH electrolyte for 1 h.

The discharge curves for the aluminum-air battery at different discharge current densities for 1 h are shown in Figure 3. It can be seen that the discharge voltage decreases with the increase in current density. The heat generation results for each part of the aluminum-air battery were obtained by discharging at different current densities.

**Figure 4:**
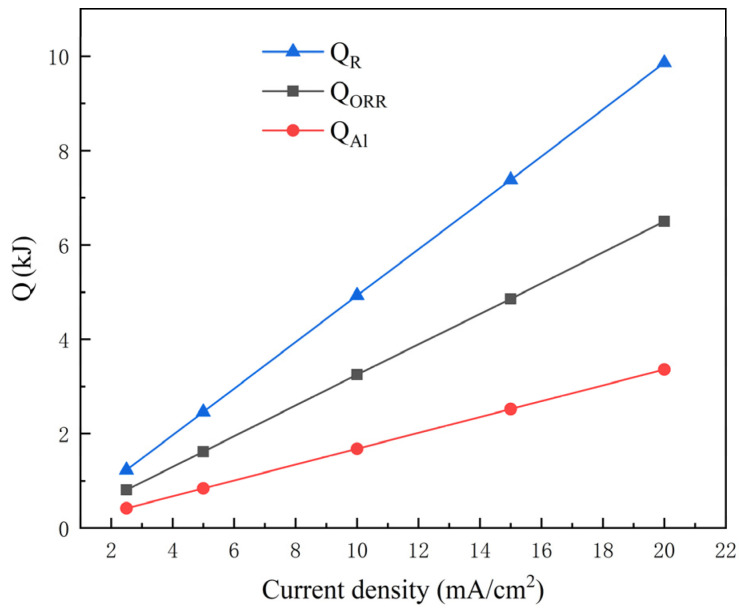
Calculation results for *Q_Al_*, *Q_ORR_*, and *Q_R_* with different discharge current densities.

*Q_R_* reflects the heat generated by the discharge reaction of the aluminum-air battery, consisting of *Q_Al_* and *Q_ORR_*; the specific values are listed in Table 2 and exhibited in Figure 4. It can be found that *Q_Al_*, *Q_ORR_*, and *Q_R_* all increased linearly with discharge current density. Obviously, fast electron transmission with a raised discharge rate was accompanied by more heat release.

**Figure 5:**
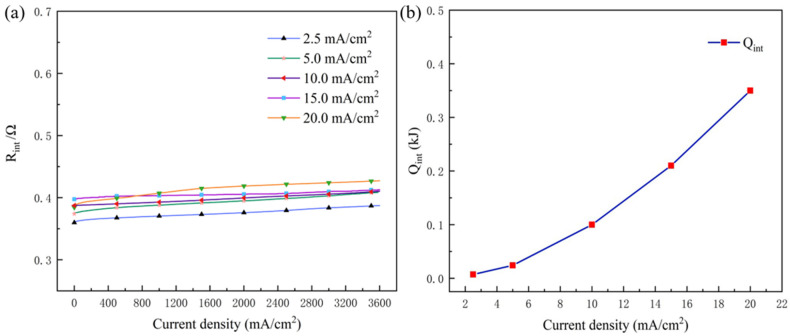
(**a**) *R_int_* for the aluminum–air battery at different current densities with discharge time and (**b**) *Q_int_* with different discharge current densities.

*Q_int_* was introduced to directly characterize the sum of *Q_J_* and *Q_P_*. The internal resistance (*R_int_*) of the battery was measured at different discharge densities, as shown in Figure 5a, and the corresponding values of *Q_int_* are exhibited in Figure 5b. It can be observed that *R_int_* increased slightly (from 0.35 and 0.4) with current density, exhibiting little change with discharge time. Therefore, the values of *Q_int_* calculated by Equation (25) are mainly determined by discharge current density.

**Figure 6:**
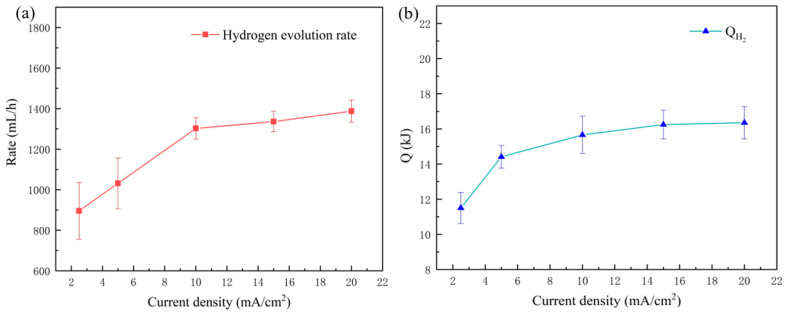
(**a**) Hydrogen-evolution rate and (**b**) *Q*_*H*_2__ with different discharge current densities.

*Q*_*H*_2__ is the heat released by the hydrogen-evolution reaction, and the corresponding evolution rate of hydrogen from the aluminum electrode was recorded during the discharge process (Figure 6a). The hydrogen-evolution rate maintained an upward tendency to 1400 mL/h with a current density of 20 mA/cm^2^. The increasing hydrogen-evolution rate slowed when the current density was greater than 10 mA/cm^2^. This observation is mainly attributed to the increase in hydrogen-evolution potential induced by aluminum electrode polarization and the resulting inhibition effect on the hydrogen-evolution reaction [25]. In accordance with Equations (28) and (31), the values for *Q*_*H*_2__ also exhibited a similar trend, as shown in Figure 6b.

**Figure 7:**
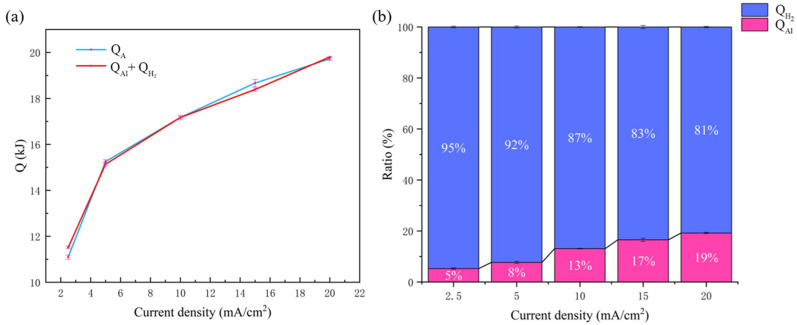
(**a**) *Q_A_* with different discharge current densities measured by direct and indirect methods and (**b**) the proportion of *Q_Al_* and *Q*_*H*_2__ in *Q_A_* of the aluminum–air battery.

The heat generated by the aluminum anode reaction is given as *Q_A_*, which consists of *Q_Al_* and *Q*_*H*_2__ and related to the heat from the discharge reaction of the aluminum electrode and the hydrogen-evolution reaction, respectively. By measuring the weight loss of the aluminum anode after discharge process (Table 3), *Q_A_* can also be directly obtained based on Equations (15) and (32), indicating good consistency with that calculated by summing *Q_Al_* and *Q*_*H*_2__, as shown in Figure 7a. Subsequently, the proportion of *Q_Al_* and *Q*_*H*_2__ was further analyzed quantitatively (Figure 7b), showing that *Q*_*H*_2__ accounted for most of the heat, up to 95%. Additionally, the ratio (*Q*_*H*_2__/*Q_A_*) decreased gradually with discharge current density, but remained greater than 80%.

According to Equation (8) and relevant analysis of *Q_R_*, *Q_J_*, and *Q_P_*, *Q_tol_* can be expressed by Equation (34):

(34) $$Q_{tol}=Q_{Al}+Q_{ORR}+Q_{int}+Q_{H_{2}}$$

**Figure 8:**
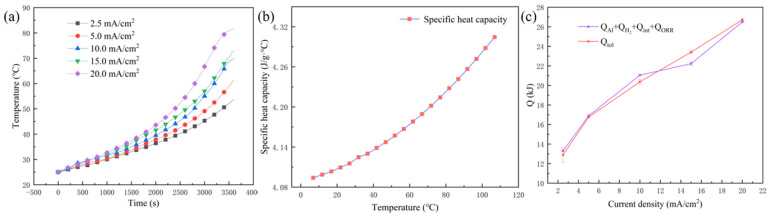
(**a**) The change in temperature of the aluminum–air battery with discharge time, (**b**) the specific heat capacity of the electrolyte with temperature, and (**c**) *Q_tol_* with different discharge current densities obtained by direct and indirect methods.

The value of *Q_tol_* can also be directly calculated based on Equation (7) by monitoring the temperature change (∆*T* listed in Table 3), which was continuously recorded during the discharge process (Figure 8a), and the specific heat capacity of electrolyte (Figure 8b). This experimental value is also in good agreement with the calculated value according to Equation (34), as illustrated in Figure 8c. It can be seen clearly that the temperature of the aluminum-air battery increased with discharge current density, as shown in Figure 7a. When the aluminum-air battery was discharged at 2.5 mA/cm^2^, the temperature increased by 28.6 °C. When the aluminum-air battery was discharged at 5.0 mA/cm^2^, the temperature increased by 36.3 °C. When the aluminum-air battery was discharged at 10.0 mA/cm^2^, the temperature increased by 44.3 °C. At 15.0 mA/cm^2^ discharge, the temperature increased by 47.8 °C. At 20.0 mA/cm^2^ discharge, the temperature increased by 56.5 °C. Thus, the larger the current density, the higher the temperature rise of the aluminum-air battery. It should be further noted that the temperature rising rate also accelerated gradually. The larger the discharge current density, the faster the temperature rose, and more easily the battery became unstable in a short period, resulting in a thermal runaway of the battery [33]. The specific values of *Q_Al_*, *Q_int_*, *Q_ORR_*, *Q*_*H*_2__, and *Q_tol_* are listed in Table 2, and the relevant proportions of each part are shown in Figure 8. It can be shown that *Q*_*H*_2__ accounted for more than 60%, especially up to 90% at relatively lower discharge rate (2.5 mA/cm^2^), resulting in the enhanced heat release. In conclusion, *Q*_*H*_2__ played the dominant role in the thermal effect during the discharge process, and effective inhibition of the hydrogen-evolution reaction is essential to avoid the thermal runaway of the aluminum-air battery.

### 3.3. Regulating the Thermal Effect of the Aluminum–Air Battery

**Figure 9:**
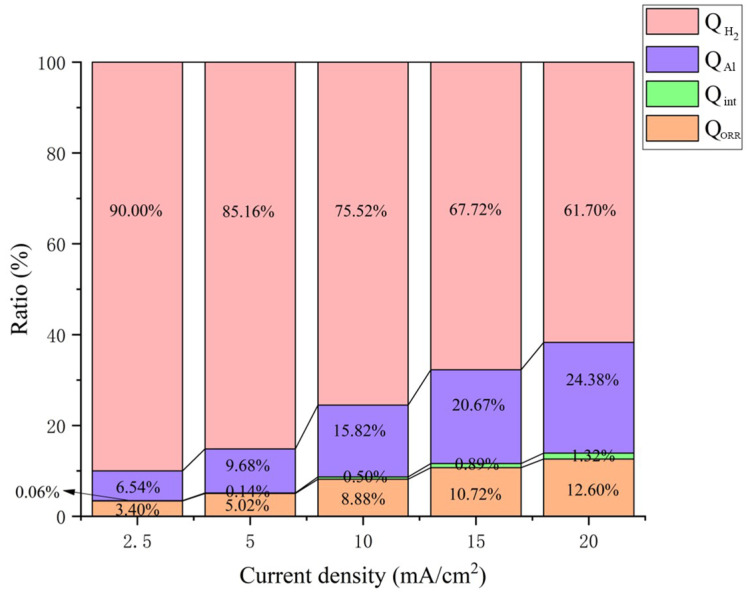
The proportion of Q_Al_, Q_ORR_, Q_int_, and *Q*_*H*_2__ in Q_tol_ under different discharge current densities.

**Figure 10:**
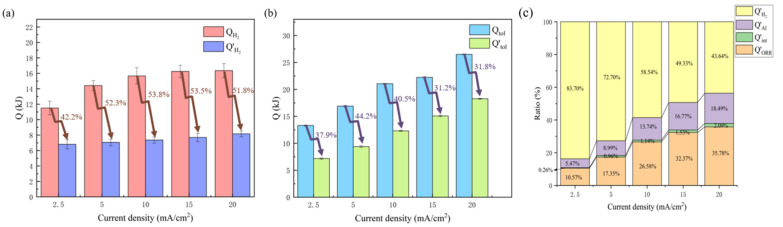
Comparison of (**a**) heat generated by hydrogen-evolution reaction, (**b**) total heat, and (**c**) the proportion of each part with modification by electrolyte additives.

It can be seen from Figure 9a that hydrogen-evolution heat generation accounted for a very large amount of the overall heat generation. A regulation strategy for inhibiting the hydrogen-evolution reaction was proposed by means of hybrid additives (0.16 M DTAB and 0.2 M ZnO) to the electrolyte. The corresponding values of *R’*_*H*_2__, *Q’_H_2__*, and *Q’_tol_* are listed in Table 4 and then compared with that free from additives, as shown in Figure 10a,b. It can be seen clearly that the hydrogen-evolution rate (*R’*_*H*_2__) of the aluminum–air battery was greatly reduced by more than 50% under different discharge current densities, and the reduction in the hydrogen-evolution rate increased with the increase in current density. When the discharge current density was 20 mA/cm^−2^, the hydrogen-evolution rate decreased by 58.4%. Hydrogen-evolution heat production was also greatly reduced by more than 50%; the maximum could be reduced by 53.8%, and the overall heat production was also reduced by more than 30%, the maximum could be reduced by 44.2%. The results showed that the hybrid addition of DTAB and ZnO could effectively reduce the heat production of the hydrogen-evolution reaction and the overall heat production, which could effectively improve the stability of the battery. In addition, the detailed proportional analyses of heat generation for each part are shown in Figure 10c. The heat-generation ratio of the hydrogen-evolution reaction decreased significantly, and the decrease in heat generation also increased with the increase in current density. This also provides an effective strategy to regulate the thermal effect and promotes the practical application of aluminum-air batteries.

## 4. Conclusions

(1) The thermal effect of an aluminum-air battery was investigated to provide a theoretical model by combining experimental measurement and mathematical calculation. The generated heat during the discharge process (*Q_tol_*) was divided into the following parts: aluminum oxidation reaction heat at the anode (*Q_Al_*), oxygen reduction reaction at the cathode (*Q_ORR_*), heat production against the battery’s internal resistance (*Q_int_*, sum of *Q_J_* and *Q_P_*), and the hydrogen-evolution heat (*Q*_*H*_2__).
(2) The heat of each part noted above was quantitatively analyzed with different discharge rates. It could be shown that all heat production increased with the increase in current density, accelerating the rise in temperature of the battery. Obviously, the battery became more unstable at high discharge rates, resulting in thermal runaway in a short period.
(3) Hydrogen-evolution heat (*Q*_*H*_2__) accounted for most of the total heat, more than 60% and up to 90% at the low discharge rate of 2.5 mA/cm^2^. It can be concluded that *Q*_*H*_2__ plays the dominant role in thermal effect during the discharge process of the battery. The hydrogen-evolution reaction should be inhibited to avoid thermal runaway of the battery.
(4) Hybrid additives of DTAB and ZnO were introduced into the electrolyte to inhibit the hydrogen-evolution reaction at the aluminum electrode. The hydrogen-evolution rate was greatly reduced, lowering its contributed heat by more than 50% and its portion in *Q_tol_*. This also provides an effective way to solve the crucial challenge of battery thermal runaway.

## Figures and Tables

**Table 1:** Thermodynamic data for ∆_f_H_m_^θ^ (298 K) and ∆C_p,m_ (B) at 298.15 K.

Substance	∆_f_H_m_^θ^ (298 K) (kJ·mol^−1^)	C_p,m_ (J·deg^−1^·mol^−1^)
Al	0	24.4
H_2_O	−285.83	37.11
OH^−^	−230.015	−148.5
H_2_	0	28.84
Al(OH)_4_^−^	−1502.5	5.12 *
Al^3+^	−538.4	3.98 *
O_2_	0	0.92

* experimental data.

**Table 2:** Heat production for each part at different discharge current densities.

Current Density/mA·cm^−1^	Q_R_/kJ	Q_Al_/kJ	Q_ORR_/kJ	Q_H_2__/kJ	Q_tol_/kJ	Q_int_/kJ	Q_A_/kJ	Q_Al_ + Q_H_2__/kJ	Q_Al_ + Q_ORR_ Q_H_2__ + Q_int_/kJ
2.5	1.23	0.42	0.81	11.13 ± 0.88	13.38 ± 0.09	0.007	11.03 ± 0.20	11.55 ± 0.88	12.36 ± 0.88
5	2.46	0.84	1.62	14.25 ± 0.64	16.98 ± 0.12	0.024	15.31 ± 0.16	15.09 ± 0.64	16.74 ± 0.64
10	4.93	1.68	3.25	15.52 ± 1.05	21.01 ± 0.06	0.10	17.24 ± 0.17	17.20 ± 1.05	20.55 ± 1.05
15	7.38	2.52	4.86	15.92 ± 0.81	22.36 ± 0.18	0.21	18.78 ± 0.24	18.44 ± 0.81	23.51 ± 0.81
20	9.86	3.36	6.50	16.45 ± 0.91	26.43 ± 0.09	0.35	19.78 ± 0.13	19.81 ± 0.91	26.66 ± 0.91

**Table 3:** The hydrogen-evolution rate (R_H_2__), internal resistance (R_int_), temperature change (∆T), and weight loss (∆m) of aluminum anode at different discharge current densities.

Current Density/mA·cm^−1^	2.5	5	10	15	20
R_H_2__/mL·cm^−2^·h^−1^	947.90 ± 81.30	1168.72 ± 78.41	1257.48 ± 52.74	1289.40 ± 50.84	1332.20 ± 54.52
R_int_/Ω	0.3752 ± 0.0121	0.3982 ± 0.0124	0.4055 ± 0.0074	0.4138 ± 0.0134	0.5063 ± 0.0296
∆T/°C	28.62 ± 2.10	36.35 ± 1.32	44.90 ± 1.89	47.80 ± 2.31	56.50 ± 1.54
∆m_Al_/g	0.55 ± 0.02	0.77 ± 0.01	0.87 ± 0.01	0.94 ± 0.03	0.99 ± 0.01

**Table 4:** The hydrogen-evolution reaction, the heat generated by the hydrogen-evolution reaction and total heat after regulating the hydrogen-evolution reaction.

Current Density/mA cm^−1^	2.5	5	10	15	20
R’_H_2__/mL·h^−1^	445.23 ± 39.30	478.76 ± 45.63	522.74 ± 34.34	552.40 ± 52.21	610.23 ± 34.52
Q’_H_2__/kJ	6.43 ± 0.59	6.79 ± 0.43	7.16 ± 0.39	7.41 ± 0.51	17.93 ± 0.38
Q’_tol_/kJ	7.68 ± 0.14	9.34 ± 0.18	12.23 ± 0.10	15.02 ± 0.14	18.17 ± 0.09

## Data Availability

Not applicable.
